# Correction: From wavelike to sub-diffusive motion: exciton dynamics and interaction in squaraine copolymers of varying length

**DOI:** 10.1039/d0sc90220a

**Published:** 2020-10-15

**Authors:** Pavel Malý, Julian Lüttig, Arthur Turkin, Jakub Dostál, Christoph Lambert, Tobias Brixner

**Affiliations:** Institut für Physikalische und Theoretische Chemie, Universität Würzburg Am Hubland 97074 Würzburg Germany brixner@phys-chemie.uni-wuerzburg.de; Institut für Organische Chemie, Universität Würzburg Am Hubland 97074 Würzburg Germany christoph.lambert@uni-wuerzburg.de; Center for Nanosystems Chemistry (CNC), Universität Würzburg Theodor-Boveri-Weg 97074 Würzburg Germany

## Abstract

Correction for ‘From wavelike to sub-diffusive motion: exciton dynamics and interaction in squaraine copolymers of varying length’ by Pavel Malý *et al.*, *Chem. Sci.*, 2020, **11**, 456–466, DOI: 10.1039/C9SC04367E.

Our original article by Malý *et al.* contains an error in the wavenumber scale of Fig. 2 and imprecisely drawn rectangles marking regions of interest for integration. The corrected version is included here.

**Fig. 2:**
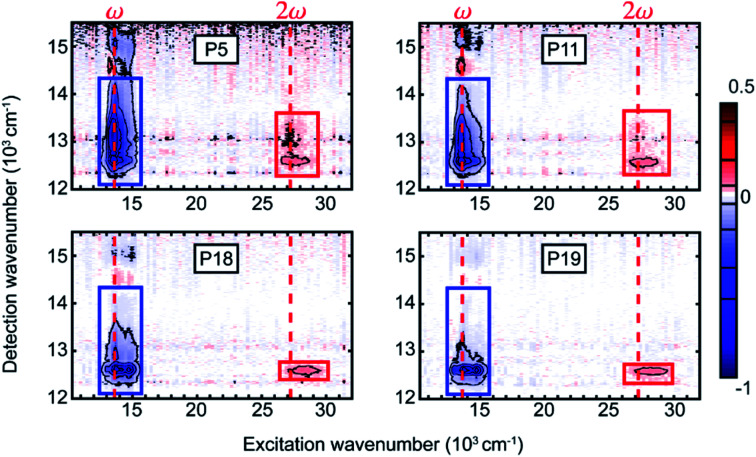
Measured two-dimensional electronic spectra for the four copolymers at population time *T* = 991 fs. We distinguish the conventional absorptive 2D (blue rectangle) and fifth-order EEI2D (red rectangle) spectra arising around the fundamental pump wavenumber or its double, respectively (red dashed lines). The rectangles indicate regions of interest for calculating integrated signals that are further analyzed as a function of the population time.

The axis distortion is only minor, and the corrections of Fig. 2 influence neither any derived data or figures, nor the further discussion, nor the conclusions of the article.

The Royal Society of Chemistry apologises for these errors and any consequent inconvenience to authors and readers.

